# Intimate relationship characteristics as determinants of HIV risk among men who have sex with regular male sex partners: a cross-sectional study in Guangzhou, China

**DOI:** 10.1186/s12879-018-3044-6

**Published:** 2018-04-02

**Authors:** Juan He, Hui-fang Xu, Wei-bin Cheng, Sheng-jie Zhang, Jing Gu, Yuan-tao Hao, Chun Hao

**Affiliations:** 1 Department of Medical Statistics and Epidemiology, School of Public Health, Sun Yat-sen University, 74 Zhongshan Road 2, Guangzhou, Guangdong 510080 People’s Republic of China; 2 Department of HIV/AIDS Control and Prevention, Guangzhou Center for Disease Control and Prevention, Guangzhou, Guangdong 510440 People’s Republic of China; 3 Current address: Medical Research & Biometrics Center, National Center for Cardiovascular Disease, Chinese Academy of Medical Science & Peking Union Medical College, A105, Xishan Institute of Fuwai Hospital, Fengcunxili, Mentougou Dist, Beijing, 102300 China; 4 Sun Yat-sen Global Health Institute, Institute of State Governance, Sun Yat-sen University, Guangzhou, Guangdong 510080 People’s Republic of China; 5 Health Information Research Center & Guangdong Key Laboratory of Medicine, School of Public Health, Sun Yat-sen University, Guangzhou, Guangdong 510080 People’s Republic of China

**Keywords:** Men who have sex with men, Intimate relationship characteristics, Unprotected anal intercourse, Interdependence theory

## Abstract

**Background:**

China faces a serious HIV epidemic among men who have sex with men (MSM), and a large proportion of new infections are attributed to their regular male sex partners (RP). The objective of this study was to investigate the association between intimate relationship characteristics and HIV-related behaviors among MSM with RP in Guangzhou, China.

**Methods:**

A convenience-sampling method was used in data collection. A total of 608 MSM were screened, of whom 406 HIV negative MSM with at least one RP in the past six months were used for data analysis. Three-step logistic regressions were used to analyze the data.

**Results:**

The prevalence of unprotected anal intercourse (UAI) with regular male sex partners, non-regular male sex partners, and concurrent UAI in the past six months was 53.9%, 23.6%, 20.7%, respectively. Variables associated with UAI with regular male sex partners included expectations for this relationship (adjusted odds ratio in multiple forward stepwise logistic regression, OR_m_ = 1.66) and open communication about the sexual relationship (OR_m_ = 1.79), while expectations for the relationship (OR_m_ = 0.46 to 0.54) and conflicts of interest (OR_m_ = 5.46 to 5.97) were associated with concurrent UAI and UAI with non-regular male sex partners.

**Conclusion:**

Intimate relationship characteristics were related to HIV-related risk behaviors. Future HIV prevention interventions should take MSM couples into consideration, include a focus on the quality of their intimate relationships, and encourage open communication about their sexual relationships.

**Electronic supplementary material:**

The online version of this article (10.1186/s12879-018-3044-6) contains supplementary material, which is available to authorized users.

## Background

China now faces a serious human HIV epidemic among men who have sex with men (MSM) [1–4], and a large proportion of new HIV infections among this population are attributed to regular male sex partners (RP) [5]. HIV prevalence among MSM in China grew from 1.4% to 7.7% from 2003 to 2014 [4, 6, 7], and new HIV infection among MSM transmitted through RP increased from 34% to 40% from 2002 to 2010 [5]. These statistics are similar to those of other countries. In the United States, 68% of HIV transmissions come from main partners, whereas only 32% come from casual male sex partners [8]. In New Zealand, 40% of new infections are transmitted by RP, with 37% of these by casual male sex partners and 23% by commercial male sex partners [9]. These statistics indicate that HIV risk behaviors occur more frequently within the context of regular or intimate relationships [10, 11]. A meta-analysis showed that the prevalence of unprotected anal intercourse (UAI) with RP among MSM in mainland China was 53%, whereas it was 45% with casual male sex partners [12]. In Hong Kong, the prevalence of UAI with RP among MSM is 60.2%, while it is 45.8% with casual male sex partners [13]. In the United States, the frequency of condom use with boyfriends among MSM is the lowest (38.6%), then almost doubling with regular sex partners other than boyfriend (61%), and is the highest with casual male sex partners (74.9%) [10]. An MSM couple-based study in the United States showed an 84% prevalence of UAI with RP, and a 66% prevalence of UAI with NRP [14]. Without the possibility of legal marriage and lacking an accepting social environment, the regular or intimate relationship among MSM is vulnerable and fluid, and thus MSM who have RP will usually have other sexual relationships outside their regular relationships and will engage in concurrent UAI [15–17]. These instances of concurrent UAI facilitate the spread of HIV among this population [18–20]. While HIV prevention programs targeting MSM made considerable efforts to promote the use of condoms with sex partners in general, MSM with regular or intimate relationships are substantially understudied [21–23].

The frequency of UAI within and outside intimate relationships among MSM is associated with the quality of the relationship [11, 24, 25]. A U.S. cross-sectional study revealed that a sexual agreement that did not allow sex with casual male sex partners (OR = 0.05) and which participants valued (OR = 0.20) could reduce UAI with both RP and NRP [26]. Another U.S. study showed that intimacy between RP could increase UAI within concordant negative relationships (OR = 1.03), but the length of the relationship (OR = 0.92) and the dependability of a partner (OR = 0.88) could reduce UAI within concordant negative relationships. This study also showed that greater attachment could increase UAI with RP both among concordant positive (OR = 1.09) and discordant (OR = 1.07) couples and open agreements that allow UAI with NRP could increase engaging in UAI with outside partners among men in concordant (OR = 9.08) and discordant (OR = 5.87) relationships [25]. In Chinese Confucian culture, MSM couples are less accepted by society and family [27]. Compared with other countries, then, MSM in China have fewer supports to maintain their intimate relationships, and MSM intimate relationships are therefore predictably more vulnerable and dynamic [28]. Consequently, MSM frequently change their RP or engage in sex with NRP [29]. Research investigating intimate relationships among this population is rare, particularly investigation into the association between intimate relationships and HIV-related risk behaviors which is now urgently warranted.

Interdependence theory (IDT) is a classic theory that describes how individuals interact. IDT can predict outcomes and is frequently applied to couple relationships [30]. In this respect, the theory emphasizes the interaction within a dyad, and it has been applied in many social science researches [24, 31, 32]. According to IDT, the behaviors of dyad can be infected by the two members’ dependence model, conflicts of interest, expectations and communications [30]. The dependence model includes the level of dependence (the degree an individual relies on his or her interaction partner), mutuality of dependence (the degree to which two members are equally dependent on one another), and the basis of dependence (the way the dependence of two persons derives from partner control or joint control) [30]. Conflicts of interest describe a situation in which the outcome can benefit member A while it may or may not benefit member B [30]. Expectations in IDT mean the expectations of members in the relationship (whether the relationship will continue for a long time and the length of the relationship). Communications relates to members’ communicating with each other during the interaction, that is, partners communicating their relevant needs, goals, and motives to each other, which is also called information-seeking [30]. These variables reflect the closeness of intimate relationships such as that of regular partners. Recently, interdependence theory has also been applied in a few MSM couples studies to investigate the risk factors of HIV-related risk behaviors [24, 25]. For example, one study showed that both in seroconcordant and serodiscordant MSM couples, those with a higher level of relationship satisfaction and commitment were less likely to engage in UAI with outside partners [24]. However, IDT-based research among MSM intimate relationships is limited, and more research is warranted.

The purpose of this study is to describe the intimate relationships of MSM based on interdependence theory, and to investigate the association between intimate relationship characteristics and HIV-related behaviors among MSM with RP in Guangzhou, China. Our hypothesis is that intimate relationship characteristics can influence UAI within and outside the intimate relationships among MSM.

## Methods

### Participants and recruitment

MSM participants were recruited during May and August of 2014 in an MSM peer-friendly HIV testing service center in Guangzhou, China. The center is a well-known LGBT community-based organization (Lingnan Fellow Health Support Center) and is co-operated by the Guangzhou Center for Disease Control and Prevention (Guangzhou CDC) [33]. Almost 80% of HIV testing among MSM in Guangzhou was completed in this service center. Eligibility criteria included men who self-reported having anal intercourse with at least one RP in the past six months, and were age 18 years or older. Those who self-reported as HIV positive were excluded since, if MSM know their HIV positive or negative status, they would change their sexual behaviors and a positive HIV status would also influence their intimate relationships. Per our study objective, MSM without a known HIV positive status comprised the target population. By examining valid associations between intimate relationships and risk sexual behaviors in this population, we could develop appropriate interventions for MSM couples with HIV unknown or negative status. The exclusion of self-reported HIV positive MSM was shared by other studies [26, 34, 35].

The convenience-sampling method was used in this study. All MSM who sought HIV testing services in this center were asked to enroll. Eligible participants were invited to a private room and informed that the questionnaire was anonymous. The questionnaire took 10 min to complete on average. Written informed consent was obtained by the experienced MSM peer staff in the center. Questionnaires were then self-administered, but participants could consult the staff if there was any confusion. The staff reviewed the questionnaire when it was completed. If there was any problem, such as missing answers or inconsistency, etc., the staff would confirm with the participant and ensure the quality of data collection.

### Ethical considerations

Verbal and written consent were provided by all participants before commencement of questionnaire completion, and to keep absolute anonymity, written consent could be signed with a nickname. Participants could quit at any time before finishing the questionnaire. The study protocol was approved by the ethics review committee of the Guangzhou Center for Disease Control and Prevention.

### Measures

#### Background characteristics

Socio-demographic characteristics (age, marital status, residence, duration of stay in Guangzhou, education, and income) and MSM-related information (sexual orientation, duration of being MSM, and recruitment venue) (Table 1) were collected in this study. Sex partnership information and UAI in the past six months were also obtained (Table 2).

**Table 1:** Demographics and MSM-related information among MSM who had regular male sex partners

	Col% (*n*)
	*N* = 406
Socio-demographic characteristics	
Age	
< 25	37.7 (153)
≥ 25	62.3 (253)
Currently married	
No	83.3 (338)
Yes	16.7 (68)
Guangzhou permanent resident	
No	59.8 (243)
Yes	40.2 (163)
Stayed in Guangzhou more than two years	
No	21.2 (86)
Yes	78.8 (320)
Higher than post-secondary education level	
No	23.6 (96)
Yes	76.4 (310)
Currently a student	
No	83.0 (337)
Yes	17.0 (69)
Monthly personal income (1000 RMB = 150 USD)	
< 4000 RMB (600 USD)	47.5 (193)
≥ 4000 RMB (600 USD)	52.5 (213)
MSM-related information	
Sexual orientation	
Bisexual/uncertain	22.7 (92)
Homosexual	77.3 (314)
Duration being MSM	
< 5 years	45.1 (183)
≥ 5 years	54.9 (223)
Male sex partners mainly recruited via	
Bar/dance hall/teahouse/club/bath/park/toilet/grassland/others	11.8 (48)
Internet/Dating apps	88.2 (358)

**Table 2:** HIV risk behaviors in the past six months among MSM who had regular male sex partners

	Col% (*n*)
	*N* = 406
Sex partnership information	
Had anal sex with multiple male sex partners	
No	43.8 (178)
Yes	56.2 (228)
Had casual male sex partner(s)	
No	50.5 (205)
Yes	49.5 (201)
Had commercial male sex partner(s)	
No	96.3 (391)
Yes	3.7 (15)
Had non-regular male sex partners (casual or commercial male sex partners)	
No	50.2 (204)
Yes	49.8 (202)
Condom use information in the past six months	
Had UAI with regular male sex partners	
No	46.1 (187)
Yes	53.9 (219)
Had UAI with NRP (casual or commercial male sex partners)	
No	76.4 (310)
Yes	23.6 (96)
Had concurrent UAI with both regular male sex partners and non-regular male sex partners	
No	79.3 (322)
Yes	20.7 (84)

#### HIV-related risk behaviors

Two types of sex partner in this study were defined: regular partner (RP) and non-regular partner (NRP). Regular partners were those the participant had sex with four times or more in the past six months, including “boyfriend,” “lover,” or “regular sex partner other than boyfriend” [10, 18, 36]. Non-regular male sex partners included casual male sex partners and commercial male sex partners. Casual male sex partners were those the participant had sex with no more than three times and had no cash or kind payment, but were not considered regular partners [10, 18, 36]. Commercial male sex partners were those who had sex with the participant by the payment of cash or kind [36]. In this study, three kinds of UAI situations in the past six months were applied as the main outcomes, including UAI with RP, UAI with NRP, and concurrent UAI. Concurrent UAI in this study was defined as having UAI with both RP and NRP in the past six months, which is usually defined as risk behaviors among MSM in relationships [18, 24, 37].

#### Intimate relationship characteristics

In accordance with different constructs of the interdependence theory [30], we asked all participants about their relationship with their RP (Table 3), which included 1) Expectations for this relationship: whether they believed this was a serious relationship (with responses of “Defining as boyfriend by each other in this relationship,” “Having not yet defined as boyfriend by each other,” “Defining as regular sex partner other than boyfriend,” and “Defining as other kind of relationship.” Those who responded as “Defining as boyfriend by each other in this relationship” were classified as “Yes”; others were classified as “No.”); Length of the relationship (with responses of “less than three months,” “three to six months,” “six or more than six months and less than 12 months,” “one to three years,” “three years or more than three years.” Those who responded as “less than three months” and “three to six months” were classified as “< 6 months”; others were classified as “≥ 6 months”) and whether they perceived this relationship would continue for a long time (with responses of “Yes” or “No”); 2) Conflicts of interest: whether they exchanged money or materials to maintain this sexual relationship (with responses of “Yes” or “No”); 3) the dependence model: whether the participant invested more emotion in this relationship (with responses of “I invested more,” “He invested more,” “We invested the same”, “None of us invested emotion in this relationship.” Those who responded as “I invested more” were classified as “Yes”; others were classified as “No.”), and whether their RP was dominant in this relationship (with responses of “I was dominant,” “He was dominant,” “We have equal power in this relationship.” Those who responded as “He was dominant” was classified as “Yes”; others were classified as “No.”); 4) Open communication in sexual relationship (openly discussed being monogamous in this relationship (with responses of “Yes” or “No”), and openly discussed a sexual agreement or requirement to use a condom when having sex with other partners (with responses of “Yes” or “No”)). In addition, we collected information about the number of RP (those who had two or more RP in the past six months were classified as “has multiple RP”).

**Table 3:** The characteristics of the relationship with regular male sex partners

	Col% (*n*)
	*N* = 406
Had multiple RP in the past six months	
No	67.5 (274)
Yes	32.5 (132)
Expectation for this relationship	
Length of the relationship	
< 6 months	52.2 (212)
≥ 6 months	47.8 (194)
Believed this was a serious relationship	
No	44.8 (182)
Yes	55.2 (224)
Perceived that this relationship wound continue for a long time	
No	20.7 (84)
Yes	79.3 (322)
Conflict of interest	
Exchanged money or materials to maintain this sexual relationship	
No	98.0 (398)
Yes	2.0 (8)
The dependence model	
Invested more emotion in this regular relationship	
No	65.3 (265)
Yes	34.7 (141)
Your RP is dominant in this relationship	
No	76.4 (310)
Yes	23.6 (96)
Open communication on sexual relationship	
Openly discussed being monogamous in this relationship	
No	60.8 (247)
Yes	39.2 (159)
Openly discussed a sexual agreement or requirement to use condoms when having sex with other partners	
No	47.5 (193)
Yes	52.5 (213)

### Statistical analysis

Except for description of data, we constructed univariate and multivariate logistic regression models to investigate the association between intimate relationship characteristics and UAI (UAI with RP, UAI with NRP and concurrent UAI) in the past six months, and we built multiple forward stepwise logistic regression models to confirm the association between intimate relationship characteristics and UAI in the past six months. A three-stage strategy was used to investigate our final objective.

First, univariate odds ratios (and 95% confidence intervals) were derived for associations between intimate relationship characteristics variables and the dependent variables (UAI with RP, UAI with NRP, and concurrent UAI in past six months).

In the second stage, after adjusting for the confounders, adjusted odds ratios (and 95% confidence interval) were applied to describe the association between intimate relationship characteristics and the dependent variables. Confounders adjusted in this stage and the final multiple forward stepwise logistic regression models were detected by universal directed acyclic graphs (DAGs). DAGs use causal diagrams to visualize the causal effects between exposures and outcomes and their use is gaining popularity in epidemiology and biostatistics [38]. Based on the aforementioned selection process, age (as a continuous variable), marital status, education level, Guangzhou permanent residency, sexual orientation, duration being MSM, and recruitment venue were treated as confounders in all multivariate and multiple forward stepwise models. Similar data analysis methods can be seen in other studies [34, 39, 40].

Last, after adjusting for the previously mentioned confounders, the independent intimate relationship characteristics variables in the previous stage were included in the final multiple forward stepwise logistic regression models. The multiple forward stepwise logistic regression model has also been used in another study [11]. In all models, statistical significance was defined by *p* value < 0.05. Data from this study was analyzed with SAS (SAS 9.1 for windows; SAS Institute Inc., NC).

## Results

Six hundred eight MSM were screened, and 526 respondents reported having had anal intercourse with men in the past six months; 412 of those respondents self-reported having had anal intercourse with at least one RP. Of those 412 respondents, six self-reported as HIV positive. After all disqualifications, 406 eligible respondents were included in the analysis.

### Socio-demographic and MSM-related information

The mean age of 406 eligible participants was 28.2 ± 6.8 years old, and 37.7% were aged younger than 25 years. Of all eligible participants, 83.3% were currently single, 40.2% were Guangzhou permanent residents, 78.8% had stayed in Guangzhou more than two years, 76.4% had obtained a post-secondary education, 17.0% were students, and 52.5% earned more than 4000 RMB (600 USD) per month. Regarding sexual orientation, 77.3% self-identified as homosexual, and 54.9% reported being MSM more than five years. A total of 88.2% participants mainly recruited sex partners via the Internet (Table 1).

### HIV-related risk behaviors in the past six months

In the past six months, 56.2% of participants had anal intercourse with multiple male sex partners and 49.8% had NRP (49.5% had casual male sex partners; 3.7% had commercial male sex partners). Among those with specific types of sex partners, the prevalence of UAI with RP and UAI with NRP was 53.9% and 23.6%, respectively, while 20.7% participants had concurrent UAI with both RP and NRP (Table 2).

### Intimate relationship characteristics

Thirty-two point 5% of 406 eligible participants had multiple RP, 47.8% said their relationship had lasted more than six months, 55.2% believed this was a serious relationship, 79.3% perceived that the relationship would continue for a long time, 2.0% had monetary or materials exchange to maintain the relationship with their RP, 34.7% thought they invested more emotion than their partners in their regular relationship, 23.6% reported that their partners were dominant in the relationship, 39.2% openly discussed with their RP being monogamous in the relationship, and 52.5% openly discussed with their RP a sexual agreement or requirement to use condoms when having sex with other partners (Table 3).

### Intimate relationship characteristics associated with UAI with regular male sex partners

Fifty-three point 9% of participants had UAI with RP in the past six months. Univariate and multivariate models detected the same risk factors of UAI with RP (Table 4). Results of these two models showed that participants who believed they were in a serious relationship had greater odds of engaging in UAI with RP (AOR = 1.75, 95% CI: 1.16–2.64, *p* < 0.01). The longer participants were in the relationship, the more likely they were to engage in UAI with RP (AOR = 1.69, 95% CI: 1.11–2.58, *p* < 0.05), and participants who had openly discussed with their RP being monogamous in this relationship were also more likely to engage in UAI with RP (AOR = 1.83, 95% CI: 1.21–2.79, *p* < 0.01) compared with those who had not openly discussed monogamy. After adjusting for confounders, the results of multiple forward stepwise logistic regression models showed that the length of the relationship (OR_m_ = 1.67, 95% CI: 1.09–2.56, *p* < 0.05) and open discussion with RP about being monogamous in the relationship (OR_m_ = 1.81, 95% CI: 1.19–2.76, *p* < 0.01) could increase the episodes of UAI with RP (Table 4).

**Table 4:** Intimate relationship characteristics associated with UAI with regular male sex partners in the past six months

Factors	UAI with regular male sex partners			
	Row% (n/n_1_)	OR (95% CI)	AOR (95% CI)	OR_m_ (95% CI)
Had multiple RP in the past six months				
No	51.5 (141/274)	1	1	
Yes	59.1 (78/132)	1.36 (0.90,2.07)	1.31 (0.85,2.03)	–
Expectation for this relationship				
Length of the relationship				
< 6 month	49.1 (104/212)	1	1	
≥ 6 month	59.3 (115/194)	1.51 (1.02,2.24)*	1.69 (1.11,2.58)*	1.67 (1.09,2.56)*
Believed this was a serious relationship				
No	46.7 (85/182)	1	1	
Yes	59.8 (134/224)	1.70 (1.14,2.52)**	1.75 (1.16,2.64)**	NS
Perceived that this relationship would continue for a long time				
No	52.4 (44/84)	1	1	
Yes	54.4 (175/322)	1.08 (0.67,1.75)	1.17 (0.71,1.96)	–
Conflict of interest				
Exchanged money or materials to maintain this sexual relationship				
No	53.5 (213/398)	1	1	
Yes	75.0 (6/8)	2.61 (0.52,13.07)	2.50 (0.49,12.82)	–
The dependence model				
Invested more emotion in this regular relationship				
No	53.6 (142/265)	1	1	
Yes	54.6 (77/141)	1.04 (0.69,1.57)	1.03 (0.68,1.57)	–
Your RP is dominant in this relationship				
No	55.5 (172/310)	1	1	
Yes	49.0 (47/96)	0.77 (0.49,1.22)	0.75 (0.46,1.22)	–
Open communication on sexual relationship				
Openly discussed being monogamous in this relationship				
No	48.2 (119/247)	1	1	
Yes	62.9 (100/159)	1.82 (1.21,2.74)**	1.83 (1.21,2.79)**	1.81 (1.19,2.76)**
Openly discussed a sexual agreement or requirement to use condoms when having sex with other partners				
No	53.9 (104/193)	1	1	
Yes	54.0 (115/213)	1.00 (0.68,1.48)	1.04 (0.70,1.56)	–

**†***p* < 0.10, *p < 0.05, **p < 0.01

*AOR* Adjusted odds ratio in multivariate logistic regression, *OR*_*m*_ Adjusted odds ratio in multiple forward stepwise regression

AOR and OR_m_ were calculated after adjusting for confounders, and confounders including age (as a continuous variable), marital status (currently married or not), Guangzhou permanent resident (Yes or No), education level (higher than post-secondary education level or not), sexual orientation (bisexual/uncertain, or homosexual), and duration being MSM (less than five years, five years/ longer than five years), recruitment via Internet (Yes or No)

n_1_: n_1_ actually is the n in Table 3, to distinguish the n in Tables 4, 5, 6, we use n_1_ to represent the n of Table 3

NS**:** Variables with *p* < 0.10 in multivariate logistic regression, but were not significant in multiple forward stepwise logistic regression

### Intimate relationship characteristics associated with UAI with non-regular male sex partners

One fourth of participants (23.6%) had UAI with NRP in the past six months. After adjusting for confounders, five intimate relationship characteristics were significantly or marginally significantly associated with UAI with NRP. Those who had multiple RP in the past six months (AOR = 1.63, 95% CI: 1.05–2.66, *p* < 0.05) were more likely to have UAI with NRP than those who had only one RP, and those who believed they were in a serious relationship (AOR = 0.67, 95% CI: 0.42–1.07, *p* < 0.1) had less UAI with NRP. Participants who perceived that this relationship would continue for a long time (AOR = 0.55, 95% CI: 0.32–0.95, *p* < 0.05) had good expectations for the relationship, and proved to have less UAI with NRP. Those who exchanged money or materials to maintain this sexual relationship (AOR = 5.76, 95% CI: 1.27–26.14, *p* < 0.05) had higher odds of UAI with NRP than those who had no such tangibles exchanged. RP who openly discussed a sexual agreement or requirement to use condoms when having sex with other partners (AOR = 0.66, 95% CI: 0.41–1.06, *p* < 0.1) were less likely to engage in UAI with NRP than those who had no such discussion. According to the results of the multiple forward stepwise logistic regression models, those who perceived that this relationship would continue for a long time (OR_m_ = 0.56, 95% CI: 0.32–0.97, *p* < 0.05) had less UAI with NRP than those who had bad expectations for their future, while participants who exchanged money or materials to maintain this sexual relationship (OR_m_ = 5.61, 95% CI: 1.22–25.74, *p* < 0.05) had more UAI with NRP than those who had no such tangibles exchanged (Table 5).

**Table 5:** Intimate relationship characteristics associated with UAI with non-regular male sex partners in the past six months

Factors	UAI with non-regular male sex partners			
	Row% (n/n_1_)	OR (95% CI)	AOR (95% CI)	OR_m_ (95% CI)
Had multiple RP in the past six months				
No	20.4 (56/274)	1	1	
Yes	30.3 (40/132)	1.69 (1.05,2.72)*	1.63 (1.00,2.66)*	NS
Expectation for this relationship				
Length of the relationship				
< 6 month	25.0 (53/212)	1	1	
≥ 6 month	22.2 (43/194)	0.85 (0.54,1.35)	0.90 (0.55,1.46)	–
Believed this was a serious relationship				
No	28.0 (51/182)	1	1	
Yes	20.1 (45/224)	0.65 (0.41,1.02)†	0.67 (0.42,1.07)†	NS
Perceived that this relationship would continue for a long time				
No	34.5 (29/84)	1	1	
Yes	20.8 (67/322)	0.50 (0.30,0.84)**2	0.55 (0.32,0.95)*	0.56 (0.32,0.97)*
Conflict of interest				
Exchanged money or materials to maintain this sexual relationship				
No	22.9 (91/398)	1	1	
Yes	62.5 (5/8)	5.62 (1.32,23.98)*	5.76 (1.27,26.14)*	5.61 (1.22,25.74)*
The dependence model				
Invested more emotion in this regular relationship				
No	21.5 (57/265)	1	1	
Yes	27.7 (39/141)	1.40 (0.87,2.24)	1.48 (0.92,2.40)	–
Your RP is dominant in this relationship				
No	23.6 (73/310)	1	1	
Yes	24.0 (23/96)	1.02 (0.60,1.75)	1.09 (0.62,1.91)	–
Open communication on sexual relationship				
Openly discussed being monogamous in this relationship				
No	26.3 (65/247)	1	1	
Yes	19.5 (31/159)	0.68 (0.42,1.10)	0.69 (0.42,1.13)	–
Openly discussed a sexual agreement or requirement to use condoms when having sex with other partners				
No	28.0 (54/193)	1	1	
Yes	19.7 (42/213)	0.63 (0.40,1.00)†	0.66 (0.41,1.06)†	NS

**†***p* < 0.10, *p < 0.05, **p < 0.01

*AOR* Adjusted odds ratio in multivariate logistic regression, *OR*_*m*_ Adjusted odds ratio in multiple forward stepwise regression

AOR and OR_m_ were calculated after adjusting for confounders, and confounders including age (as a continuous variable), marital status (currently married or not), Guangzhou permanent resident (Yes or No), education level (higher than post-secondary education level or not), sexual orientation (bisexual/uncertain, or homosexual), and duration being MSM (less than five years, five years/ longer than five years), recruitment via Internet (Yes or No)

n_1_: n_1_ actually is the n in Table 3, to distinguish the n in Tables 4, 5, 6, we use n_1_ to represent the n of Table 3

NS**:** Variables with *p* < 0.10 in multivariate logistic regression, but were not significant in multiple forward stepwise logistic regression

### Intimate relationship characteristics associated with concurrent UAI

Twenty point 7% of participants had concurrent UAI in the past six months. After adjusting for the aforementioned confounders, three intimate relationship characteristics were significantly or marginally significantly associated with concurrent UAI. Participants who had multiple RP in the past six months (AOR = 1.67, 95% CI: 1.00–2.79, *p* < 0.1) were marginally significantly likely to have more opportunities to engage in concurrent UAI than those who had only one RP. Those who perceived that their relationship would continue for a long time (AOR = 0.49, 95% CI: 0.27–0.87, *p* < 0.05) had less chance to engage in concurrent UAI, and those who exchanged money or materials to maintain this sexual relationship (AOR = 5.98, 95% CI: 1.31–27.22, *p* < 0.05) significantly had more concurrent UAI than those who had no such tangibles exchanged. In the multiple forward stepwise logistic regression models, variable that perceived that this relationship would continue for a long time (OR_m_ = 0.49, 95% CI: 0.27–0.87, *p* < 0.05) significantly decreased the episodes of concurrent UAI, while variable that exchange of money or materials to maintain this sexual relationship (OR_m_ = 5.90, 95% CI: 1.27–27.43, *p* < 0.05) significantly increased the episodes of concurrent UAI (Table 6).

**Table 6:** Intimate relationship characteristics associated with concurrent UAI in the past six months

Factors	Concurrent UAI (had UAI with both regular male sex partners and non-regular male sex partners)			
	Row% (n/n_1_)	OR (95% CI)	AOR (95% CI)	OR_m_ (95% CI)
Had multiple RP in the past six months				
No	17.5 (48/274)	1	1	
Yes	27.3 (36/132)	1.77 (1.08,2.89)*	1.67 (1.00,2.79)†	NS
Expectation for this relationship				
Length of the relationship				
< 6 month	21.2 (45/212)	1	1	
≥ 6 month	20.1 (39/194)	0.93 (0.58,1.51)	0.95 (0.56,1.59)	–
Believed this was a serious relationship				
No	24.7 (45/182)	1	1	
Yes	17.4 (39/224)	0.64 (0.40,1.04)†	0.66 (0.40,1.10)	–
Perceived that this relationship would continue for a long time				
No	32.1 (27/84)	1	1	
Yes	17.7 (57/322)	0.45 (0.26,0.78)**	0.49 (0.27,0.87)*	0.49 (0.27,0.88)*
Conflict of interest				
Exchanged money or materials to maintain this sexual relationship				
No	19.8 (79/398)	1	1	
Yes	62.5 (5/8)	6.73 (1.58,28.76)*	5.98 (1.31,27.22)*	5.90 (1.27,27.43)*
The dependence model				
Invested more emotion in this regular relationship				
No	19.2 (51/265)	1	1	
Yes	23.4 (33/141)	1.28 (0.78,2.10)	1.42 (0.85,2.38)	–
Your RP is dominant in this relationship				
No	21.6 (67/310)	1	1	
Yes	17.7 (17/96)	0.78 (0.43,1.41)	0.87 (0.47,1.61)	–
Open communication on sexual relationship				
Openly discussed being monogamous in this relationship				
No	22.3 (55/247)	1	1	
Yes	18.2 (29/159)	0.78 (0.47,1.29)	0.81 (0.48,1.37)	–
Openly discussed a sexual agreement or requirement to use condoms when having sex with other partners				
No	24.4 (47/193)	1	1	
Yes	17.4 (37/213)	0.65 (0.40,1.06)†	0.70 (0.42,1.15)	–

**†***p* < 0.10, *p < 0.05, **p < 0.01

*AOR* Adjusted odds ratio in multivariate logistic regression, *OR*_*m*_ Adjusted odds ratio in multiple forward stepwise regression

AOR and OR_m_ were calculated after adjusting for confounders, and confounders including age (as a continuous variable), marital status (currently married or not), Guangzhou permanent resident (Yes or No), education level (higher than post-secondary education level or not), sexual orientation (bisexual/uncertain, or homosexual), and duration being MSM (less than five years, five years/ longer than five years), recruitment via Internet (Yes or No)

n_1_: n_1_ actually is the n in Table 3, to distinguish the n in Tables 4, 5, 6, we use n_1_ to represent the n of Table 3

*NS* Variables with *p* < 0.10 in multivariate logistic regression, but were not significant in multiple forward stepwise logistic regression

## Discussion

This is one of the first studies investigating the association between intimate relationship characteristics and HIV-related risk behaviors among MSM who have RP in China. This study showed that sexual risk behaviors happened frequently outside intimate relationships among MSM. Expectations for the relationship, conflicts of interest, and open communication on sexual behaviors with RP were associated with UAI within and outside the intimate relationship, whereas the dependence model was not associated with any high risk sexual behaviors.

First, our findings identified a high concurrent UAI prevalence within and outside intimate relationships among MSM. The data showed that 56.2% of MSM with RP had multiple male sex partners, which indicates that half of MSM had concurrent partnerships. The result was similar to that of other published research in China [16], and the prevalence of concurrency is also similar to the data reported in the United States (45% to 63.2%) [41–43]. Especially in this study, a quarter (23.6%) of MSM had UAI outside their regular relationship, and one fifth (20.7%) of MSM practiced the six-month period concurrent UAI. Concurrent UAI prevalence among MSM in China is relatively higher than that in the United States which is around 16% [41, 44]. However, it should be noted that concurrent UAI in this study indicated a 6-month window, and that this study did not confirm the overlapping periods of UAI with more than one partner, which could include a few participants who practiced serial monogamy in the past six months such as having UAI with a casual partner/a new regular partner after breaking up with a boyfriend within the six month period. For this reason most likely, the rate was relatively higher than that in the United States. It is well known that concurrent UAI will contribute to HIV transmission. Our results suggested that MSM in China have a high possibility of becoming infected by their RP. A previous cohort study also showed that UAI with RP, but not UAI with casual male sex partners, was the predictor of HIV seroconversion among MSM [45]. Trust is often mentioned as a reason for not using condoms with RP [35, 46–48]. It has been estimated that in the United States 68% of HIV transmissions come from main partners, whereas only 32% come from casual male sex partners [8]. Many MSM in China still think that UAI with a regular partner is safe, and they have not recognized the risk of UAI within regular relationships, even though, as we mentioned before, more HIV infection is transmitted by RP. Thus a great deal needs to be done to educate MSM on HIV-related risk behaviors related to their main partners and to make them aware of potential risks. Sexual intercourse involves at least two people, so the interpersonal process of an intimate relationship and a sexual-related discussion on behaviors among MSM couples warranted investigation to develop MSM couple-based interventions as a first and necessary step.

Second, the factors related to expectations for the intimate relationship were positively associated with risk-taking with RP. In contrast, they were negatively associated with risk-taking with NRP. These factors comprise the aspect of the perception of relationship quality and commitment, and they remained in the final models. These results were similar to findings in the United States [24, 35], but this is the first such report in China. A mixed method study among male couples showed that these couples expressed love, trust, and commitment as the most frequent reasons for not using a condom with a partner [49]. Stark et al. found that commitment was positively associated with UAI compared with not engaging in UAI with regular partners [50]. Another cohort study among MSM couples revealed that with higher levels of positive relationship dynamics (e.g., commitment, satisfaction), RP were less likely to engage in UAI with NRP [24]. It is reasonable to assume that MSM having a higher quality relationship and who value the commitment of the relationship would have less UAI outside the relationship and thus fewer risk behaviors. In a similar way, it is difficult for heterosexual couples generally to maintain a high quality relationship. However, compared with heterosexual couples, the relationship of male couples is particularly vulnerable and fluid in China due to the unacceptable social environment of a thousand-year-old traditional culture. Changing the social environment for MSM couples will take time. From the perspective of HIV prevention, coaching male couples on how to maintain a quality relationship would be a novel approach to prevent intra-dyadic and extra-dyadic HIV transmission among MSM.

Although open communication in a sexual relationship, including open discussion about being monogamous and a sexual agreement, did not remain in the final models, the results from the adjusted OR revealed these factors were positively associated with UAI within relationships and negatively associated with UAI outside relationships. Mitchell’s study [26], which was conducted among HIV-negative gay couples in the United States, also found that MSM were less likely to have UAI outside their relationship if having a sexual agreement in place that did not allow sex outside the relationship. According to these findings, open communication about monogamy and a sexual agreement between partners may improve condom use with outside partners among MSM. Couple-based HIV counseling and testing could be considered as the appropriate intervention, providing the opportunity to discuss the sexual agreement under guidance of experienced counselors. This has been proven to be effective in the potential decrease of HIV incidence among heterosexual couples in Africa [51, 52], and it has also proven to be acceptable and safe among MSM in America [21, 23]. Since few studies in China have focused on this aspect of HIV prevention and because there is an apparent lack of awareness existing among MSM, the development of interventions to promote open communication skills among MSM couples, such as couple-based HIV testing and counseling, is needed in China.

Our findings suggested that compared with MSM whose intimate relationships had no monetary or materials exchanged with RP, MSM who had monetary or materials exchanged with RP were almost six times more likely to have UAI with NRP or have concurrent UAI. According to our literature review, few studies reported an association between HIV risk behaviors and tangibles exchanged within an intimate relationship. It is possible that two individuals are not in equal power or control within a relationship if there is an exchange of monetary or materials, and this exchange may undermine the quality of their relationship. Compared with subjective variables such as believing this was a serious relationship and the perception that the relationship would continue for a long time, etc., monetary or materials exchange might function as a sensitive indicator of the quality of relationships.

Another finding was that the dependence model was not associated with HIV-risk behaviors among MSM who had RP. Very few studies reported on the dependence model among this population. We found that only one study conducted among MSM couples reported that couples with a higher degree of emotional attachment were more likely to have UAI with their RP [24]. The association between dependence models and risk behaviors among MSM who are in intimate relationships should be investigated. More research is required.

## Limitations

The results from this study should be viewed in light of several limitations, many of which are shared by other studies in this area of research. Only one individual within the intimate relationship was recruited [32, 34, 53]. Also, participants were convenience-sample recruited from an HIV testing service clinic, which is a recruitment method for a hidden population [35, 54, 55]. In addition, all measures of sexual behaviors were based on self-reporting but efforts were made to minimize bias (e.g., anonymity, training). Our study also excluded those who self-reported being HIV positive MSM, and the potential confounding effect of this variable was not able be analyzed [26, 34, 35]. Additionally, we used categorical variables to describe intimate relationships [25, 35]; these variables were not as effective as scales, and IDT-related scales [56, 57] will be considered in our future study. Another limitation is that, as mentioned above, the concurrent UAI in this study means participants had UAI with both RP and NRP in the past six months, and the overlapping periods of UAI with more than one partner was not confirmed. The rate of concurrent UAI might include those in serial monogamy within a 6-month window. Future studies should differentiate these two situations, even though the risk of transmitting HIV is similar. Finally, this is a cross-sectional study, and causal relationships cannot be inferred.

## Conclusion

Despite limitations, this study advanced the understanding of the effects of intimate relationship characteristics on UAI with specific partners among MSM with RP in China. This study highlighted a previous finding from extra-national research that the quality of an intimate MSM relationship and open communication between dyadic individuals within the relationship would influence HIV-related risk behaviors among MSM. Future longitudinal research on MSM couples is warranted to understand relationship dynamics, sexual health needs, risk for HIV and other sexually transmitted infections over time. This study also shed light on the potential for couple-based HIV prevention intervention such as couple-based counseling and testing among MSM in China, but more relevant evidence is required.

## Additional file

Additional file 1: Dataset. Intimate relationship and UAI among MSM in Guangzhou, China. (XLS 177 kb)

## Abbreviations

AOR: Adjusted odds ratio
HIV: Human immunodeficiency virus
MSM: Men who have sex with men
NRP: Non-regular male sex partner
OR: Odds ratio
ORm: Odds ratios in multiple forward stepwise logistic regression model
RP: Regular male sex partner
UAI: Unprotected anal intercourse
